# Do Older Professional Musicians Have Cognitive Advantages?

**DOI:** 10.1371/journal.pone.0071630

**Published:** 2013-08-07

**Authors:** Tarek Amer, Beste Kalender, Lynn Hasher, Sandra E. Trehub, Yukwal Wong

**Affiliations:** 1 Department of Psychology, University of Toronto, Toronto, Ontario, Canada; 2 Rotman Research Institute, Toronto, Ontario, Canada; UNLV, United States of America

## Abstract

The current study investigates whether long-term music training and practice are associated with enhancement of general cognitive abilities in late middle-aged to older adults. Professional musicians and non-musicians who were matched on age, education, vocabulary, and general health were compared on a near-transfer task involving auditory processing and on far-transfer tasks that measured spatial span and aspects of cognitive control. Musicians outperformed non-musicians on the near-transfer task, on most but not all of the far-transfer tasks, and on a composite measure of cognitive control. The results suggest that sustained music training or involvement is associated with improved aspects of cognitive functioning in older adults.

## Introduction

Recent research suggests that a lifetime of using two or more languages can result in preserved cognitive functioning in old age [1]–[3]. Bilingualism or multilingualism has also been linked to delays in the onset of Alzheimer’s disease [4]–[6]. The benefits of bilingualism are thought to be attributable to enhancement or preservation of a domain-general conflict management or cognitive control system that is recruited to resolve competition arising from two active language systems [7]. These findings encourage the search for other types of stimulating experiences that can enhance cognitive control and contribute to cognitive reserve [8] and general cognitive functioning in older adults.

One candidate for such experiences is music training and expertise. High levels of music performance require control over the focus of attention [9], integration of sensory and motor information [10], and careful planning and monitoring of performance [11]. Skilled music-making and the use of more than one language may be similar in engaging and enhancing general cognitive control mechanisms such as selective attention and conflict resolution. Bialystok and DePape [12], for example, demonstrated that music training and bilingualism have comparable benefits for young adults on some cognitive tasks. Furthermore, bilingualism [13] and music experience [14]–[15] have both been shown to be associated with enhancements in subcortical processing of auditory stimuli in noisy environments, which is correlated with improved cognitive control. Neuroanatomical and neurophysiological differences between musicians and non-musicians have been observed in auditory and sensorimotor areas that are relevant to music processing [16]–[17] and in areas of the frontal cortex related to attention regulation [18]–[19]. Neural changes accompanying music training during childhood have been shown to persist in adulthood, even after training has ceased for approximately 7 years [20]. There is ample evidence of the benefits of music training for a wide range of auditory processing tasks [21]–[22] that involve transfer to similar tasks (near transfer). The available evidence also suggests possible far-transfer effects involving working memory, attention regulation, and conflict resolution in the context of non-auditory tasks.

With respect to far transfer, music training in children has been linked to enhanced mathematical and visuospatial skills [23]–[24] (but see Forgeard, Winner, Norton, & Schlaug, 2008 [25]), short-term and working memory [26]–[27], IQ and academic ability [28] as well as vocabulary and reading [25], [29]–[30]. In addition to the correlational evidence linking music training to non-musical abilities, there is experimental evidence that music training leads to improvements in children’s non-musical abilities. For example, 6-year-old children who were assigned randomly to 36 weeks of group music lessons had greater increases in IQ than children who were assigned to group drama lessons or no lessons [31]. Similarly, 4- to 6-year-old children who completed a 20-day program of music training (approximately 2 hours daily) showed better performance on vocabulary and executive function tasks than children who completed a comparable program of visual art training [32].

Less is known about relations between music training and cognitive abilities in later adulthood. Older amateur musicians outperform non-musicians on a variety of near-transfer tasks, including speech perception in noise, gap detection and mistuned harmonic detection in auditory stimuli, and auditory working memory [33]–[34]. Evidence of far transfer has been less consistent. In some cases, older adults with high levels of music training have performed better than their musically untrained counterparts on cognitive control tasks involving nonverbal memory and cognitive flexibility [35], but the findings have been inconsistent [36]. Intensive piano lessons for older adults over the course of 6 months resulted in enhanced cognitive flexibility (Trails B of the Trail-Making Test), general processing speed, and working memory (Digit-Symbol Coding Test) relative to an untrained control group [37]. However, an early correlational study demonstrated preservation of music-related speeded motor abilities, but not general processing speed, in older expert pianists [38]. Finally, adults 75 years and older who played a musical instrument frequently were less likely to develop dementia during a 5-year follow-up than those who played infrequently or not at all [39]. The protective effect of playing a musical instrument was greater than that of other activities such as reading or doing crossword puzzles.

There are suggestions, then, that music training can have broad transfer effects in unrelated domains, resulting in superior cognitive functioning in musically skilled older adults. However, the relative paucity of studies, uncertainty about the cognitive domains that may be enhanced by music, failures to replicate some of the findings, and the theoretical and practical implications of this issue invite further study. Here we report data on late middle-aged to older professional musicians and non-musicians with similar general education, vocabulary, and overall health. We compared performance of the two groups on a near-transfer task involving pitch identification and auditory conflict resolution, on several far-transfer tasks involving visuospatial memory span and cognitive control processes (conflict resolution, resistance to distraction, and response inhibition), and on a composite measure of cognitive control. Research with young and middle-aged amateur and professional musicians has shown enhancement on far-transfer tasks involving visuospatial function and cognitive control [12], [19], [40]–[41]. On the basis of enhanced auditory processing and working memory in older amateur musicians [33]–[34], we expected late middle-aged to older professional musicians to perform better than non-musicians on the near-transfer task. On the basis of suggestive evidence of far transfer in older amateur musicians [35]–[37] and possible similarities in cognitive demands between music experience and bilingualism [12]–[13], we expected our professional musicians to perform better than non-musicians on the far-transfer tasks, particularly those assessing cognitive control.

## Method

### Participants

The participants were 43 late middle-aged to older adults from Toronto, 19 of whom were professional musicians and 24 non-musicians. All participants gave their written informed consent to participate in the study. One musicians’ overall performance on the tasks, as measured by the composite cognitive control score (see below), was almost three standard deviations below the group mean. The participant was removed from the subsequent analyses, resulting in a total of 42 participants (50 to 77 years, *M* = 60.12 years, *SD* = 6.77), 18 of whom were musicians and 24 non-musicians. Demographic information on the musicians and non-musicians is presented in Table 1. The musicians (50 to 77 years, *M = *59.17 years, *SD = *7.11) included instrumentalists (*N* = 12) who played one or more instruments (piano, clarinet, French horn, bassoon, cello, violin, organ, harpsichord, guitar, trumpet, recorder) and vocalists (*N* = 6), all but one of whom played one or more instruments. All musicians had extensive formal training (*M = *19.78 years, *SD* = 10.90) that began in childhood (*M* = 8.33 years, *SD* = 4.51), with the exception of one participant who started formal training at young adulthood. At the time of testing, they were earning at least part of their income from music performance. The non-musicians (52 to 76 years, *M* = 60.83 years, *SD* = 6.56) had minimal or no music education outside of school and did not sing regularly or play an instrument. All participants were in good general health, had normal color vision, normal or corrected-to-normal visual acuity, and normal hearing, according to self-report. Five participants in each group spoke more than one language, but English was their dominant language for speaking, reading, and writing. Three of those participants per group simultaneously learned more than one language at an early age, but English was the only language used regularly. All experimental protocols were reviewed and approved by the ethics committee of the University of Toronto.

**Table 1:** Characteristics of musicians and non-musicians.

	Musicians		Non-musicians	
Variable	*M*	*SD*	*M*	*SD*
Age (years)	59.17	7.11	60.83	6.56
Musical experience (years)	19.78	10.90	1.79	2.28
Education (years)	19.00	2.75	18.12	4.11
Shipley vocabulary (max = 40)	36.68	2.40	36.31	3.45

### Materials and Procedure

A background questionnaire requested information about total years of education, language use, general health (including vision and hearing), and music experience. Participants were also given the vocabulary section of the Shipley Institute of Living Scale (SILS) [42] to provide an index of verbal ability that is correlated with IQ [42]–[43]. Participants then completed five tasks in one session in fixed order, as indicated below.

#### Auditory stroop task

The Hamers and Lambert [44] task, as adapted by Bialystok and DePape [12], provided a measure of pitch and word identification speed as well as measures of auditory conflict resolution. Participants’ task was to indicate whether the pitch of an auditory signal was high (by pressing the “P” key, labeled “HIGH”) or low (pressing the “Q” key, labeled “LOW”). There were two control conditions. In the pitch-control condition, a high-pitched (730.47 Hz) or low-pitched (180.44 Hz) sound, *ahh,* was presented on every trial. In the word-control condition, the word “high” or “low” was presented on every trial. There were two conflict conditions in which the words “high” and “low” were presented at a high (753.56 Hz) or low (180.13 Hz) pitch level. The congruent trials had matching words and pitch level (e.g., “high” spoken with high pitch), and the incongruent trials had mismatching words and pitch level (e.g., “high” spoken with low pitch). In the pitch-conflict condition, participants indicated the pitch of the stimulus, irrespective of the word, by pressing the “HIGH” key for a high-pitched stimulus and the “LOW” key for a low-pitched stimulus. In the word-conflict condition, participants indicated the word, irrespective of its pitch, by pressing the “HIGH” key for the word “high” and the “LOW” key for the word “low”. Stimuli were presented at a listening volume comfortable for each participant, as per each participant’s report.

Each condition was preceded by 16 practice trials, and the order of conditions was varied randomly across participants. Each trial began with a central fixation cross which remained on the screen until a response was registered. The auditory stimulus, which was presented via headphones 250 ms after the onset of the fixation cross, was 550 ms in duration. There were 48 trials for each of the pitch and word control conditions, with high and low stimuli presented randomly. There were 96 trials for each of the conflict conditions, with an equal number (48) of congruent and incongruent trials presented randomly. Reaction time (RT) and response accuracy were recorded, and trials with errors were excluded from the RT analysis. The measure of conflict resolution (i.e., the Stroop Effect) is the increase in reaction time for responding on incongruent than on congruent trials. Equipment error resulted in missing data for one non-musician.

#### Simon task

This task, another measure of conflict resolution, was adapted from Simon and Rudell [45]. Participants were required to indicate whether a 2 cm×2 cm square, which appeared on the left or right side of the computer screen, was red or blue by pressing the L key for red and the A key for blue (keys were color coded). On half of the trials (congruent condition), the square was on the same side as the response key, and on the other half (incongruent condition), the square was on the opposite side of the screen relative to the response key. The task began with 8 practice trials followed by 28 test trials. Each trial began with a 250- ms fixation cross in the center of the screen, followed by a colored square stimulus that appeared until a response occurred or 550 ms had elapsed. Reaction time and response accuracy were recorded on each trial, and error trials were excluded from the RT analysis. The Simon Effect is the increase in RT for responding on incongruent trials than on congruent trials.

#### Visuospatial span task

This task, which was adapted from Rowe, Hasher, and Turcotte [46], is a computerized version of the Corsi Block-tapping Test (CBT) [47] that measures the span of visual working memory. Nine two-dimensional gray squares (3 cm×3 cm), arranged spatially as in Corsi [47], were presented against a white background. On every trial, some gray squares turned black briefly (1000 ms) and successively. Participants were required to recall the original locations in their order of presentation. Participants responded, without time limits, using a computer mouse to click on each square sequentially. The sequences were those used in the spatial span task of the Wechsler Memory Scale – Third Edition [48]. They were presented in ascending order of difficulty, starting with a set size of four squares and ending with a set size of seven squares. For every set size, there were 3 trials, for an overall total of 12 trials. Six practice trials consisting of two- and three-square sequences were administered first to familiarize participants with the procedure. Responses were recorded automatically on each trial, and the percentage of correctly recalled sequences provided an index of visuospatial working-memory span. Experimenter error resulted in missing data from one musician.

#### Go/No-Go

This task was used to test inhibitory control over prepotent responses [49]. In the task, X’s and O’s were presented sequentially in the center of the screen for 200 ms each, with 900-ms inter-stimulus intervals (ISIs). Participants were instructed to respond to all “O”s (Go trials) by pressing the space bar and to withhold their responses whenever an “X” (No-Go trial) was presented. The task began with 30 practice trials (20 Go and 10 No-Go, intermixed) followed by 200 test trials (150 Go, 50 No-Go). The trials were presented in random order with the restriction of no consecutive No-Go trials. Reaction times on Go trials and the number of responses on No-Go trials (i.e., false alarms) were recorded. Reaction time indicated speed of responding, and the number of false alarms provided an index of the ability to inhibit prepotent responses.

#### Reading with distraction

This task, adapted from Connelly, Hasher, and Zacks [50], was used to test control over concurrent distraction [49], [51]. The task consisted of two conditions: a low-interference condition and a high-interference condition, with the latter administered first. In each condition, participants were instructed to read aloud a narrative passage (e.g., about a student going on an archaeology dig) at a comfortable pace. To encourage close attention to the stories, participants were told that they would be asked a few questions about the content. The relevant or target text in each paragraph was italicized and interspersed with irrelevant, non-italicized text. In the low-interference condition, which consisted of two stories, the irrelevant text was a string of X’s. In the high-interference condition, which consisted of four stories, the irrelevant text consisted of words or phrases related to the story content. Reading time in each condition, the number of omitted target words, and the number of distractors read (i.e., false alarms or intrusions) were recorded. These measures provided an index of participants’ ability to control irrelevant information. Data from one non-musician were excluded because of failure to follow task instructions.

## Results

The only demographic variable that reliably differentiated musicians from non-musicians was the number of years of music education, *t*(40) = 7.89, *p*<.0001, *d* = 2.46, with the non-musicians having little or no music education. Table 2 presents means and standard deviations (*SD*s) for all dependent variables. Reaction time cut-offs were two *SD*s beyond the mean for each participant. Unless otherwise noted, comparisons of musicians and non-musicians were conducted with 2×2 mixed-model analyses of variance (ANOVAs). Because accuracy scores on the Auditory Stroop and Simon tasks were not normally distributed, nonparametric Mann-Whitney tests were used to examine differences between the two groups.

**Table 2:** Mean scores and standard deviations for musicians and non-musicians.

		Musicians		Non-musicians	
Task		*M*	*SD*	*M*	*SD*
Auditory Stroop RT					
Control Conditions					
Pitch (ms)		480	69	592	134
Word (ms)		566	70	577	75
Pitch Conflict					
Congruent (ms)		628	91	764	148
Incongruent (ms)		722	125	914	184
Stroop Effect (ms)		94	90	150	83
Word Conflict					
Congruent (ms)		623	82	615	99
Incongruent (ms)		691	88	660	82
Stroop Effect (ms)		68	45	45	50
Simon Task RT					
Congruent (ms)		509	84	518	94
Incongruent (ms)		538	68	614	103
Simon Effect (ms)		30	52	97	62
Visuospatial Span (% correct)		74.06	14.64	61.54	13.68
Go/No-Go					
Go RTs (ms)		333	50	346	34
False Alarms		3.67	3.74	5.71	4.99
Reading with Distraction					
Low-interference (s)		66.19	10.73	72.15	14.35
High-interference (s)		92.50	18.89	109.12	29.86
Difference (s)		26.31	13.56	36.97	19.13
Target Words Missed		2.67	3.60	6.78	7.02
Intrusions		2.89	3.60	5.26	3.82

### Auditory Stroop Task

RT comparisons for the two control conditions (pitch and word identification) showed main effects of Condition, *F* (1, 39) = 5.12, *p*<.05, η*_p_* ^2^ = .12, and Group, *F* (1, 39) = 6.23, *p*<.05, η*_p_* ^2^ = .14, which were qualified by a significant interaction between Condition and Group, *F* (1, 39) = 10.19, *p*<.005, η*_p_* ^2^ = .21. The groups did not differ in speed of identifying a word, *p*>.6, but musicians were faster than non-musicians at identifying the relative pitch level (high or low) of signals, *t*(39) = 3.24, *p*<.005, *d* = 1.02. Musicians were also faster at identifying pitch than words, *t*(17) = 7.73, *p*<.0001, *d* = 1.24, but RTs in non-musicians did not differ between pitch or word identification, *p*>.5. Musicians (*Mdn* = 100%) were more accurate than non-musicians (*Mdn* = 98%) on the pitch-control condition, *U* = 119.50, *z* = 2.63, *p*<.01, and on the word-control condition (musicians: *Mdn = *100%; non-musicians: *Mdn* = 98%), *U* = 126.50, *z* = 2.51, *p*<.05.

RT comparisons for the pitch-conflict congruent and incongruent trials showed main effects of Congruency, *F* (1, 39) = 81.70, *p*<.0001, η*_p_* ^2^ = .68, and Group, *F* (1, 39) = 14.21, *p*<.005, η*_p_* ^2^ = .27, and a significant interaction, *F* (1, 39) = 4.25, *p*<.05, η*_p_* ^2^ = .10. As expected, performance was faster on congruent than on incongruent trials, and musicians were faster than non-musicians on the congruent, *t*(39) = 3.42, *p*<.005, *d* = 1.08, and incongruent, *t*(39) = 3.78, *p*<.001, *d* = 1.19, trials. Additionally, musicians showed a significantly smaller pitch-conflict Stroop effect, *t*(39) = 2.06, *p*<.05, *d* = .65. Musicians (*Mdn* = 99%) were significantly more accurate than non-musicians (*Mdn* = 97%), *U* = 73.50, *z* = 3.61, *p*<.0005.

RT comparisons for the word-conflict congruent and incongruent trials showed a main effect of Congruency, *F* (1, 39) = 56.09, *p*<.0001, η*_p_* ^2^ = .59, but no effect of Group, *F* <1, or interaction, *F* (1, 39) = 2.26, *p* = .14, η*_p_* ^2^ = .06. Thus, musicians and non-musicians showed a reliable word-conflict Stroop effect, with the effect being numerically, but not significantly, smaller for non-musicians. In effect, it was somewhat easier for non-musicians than musicians to ignore pitch information when identifying words, consistent with their slower identification of pitch in the control condition. Accuracy for both groups (*Mdn* = 99%) did not differ, *p*>.7.

**Figure 1:**
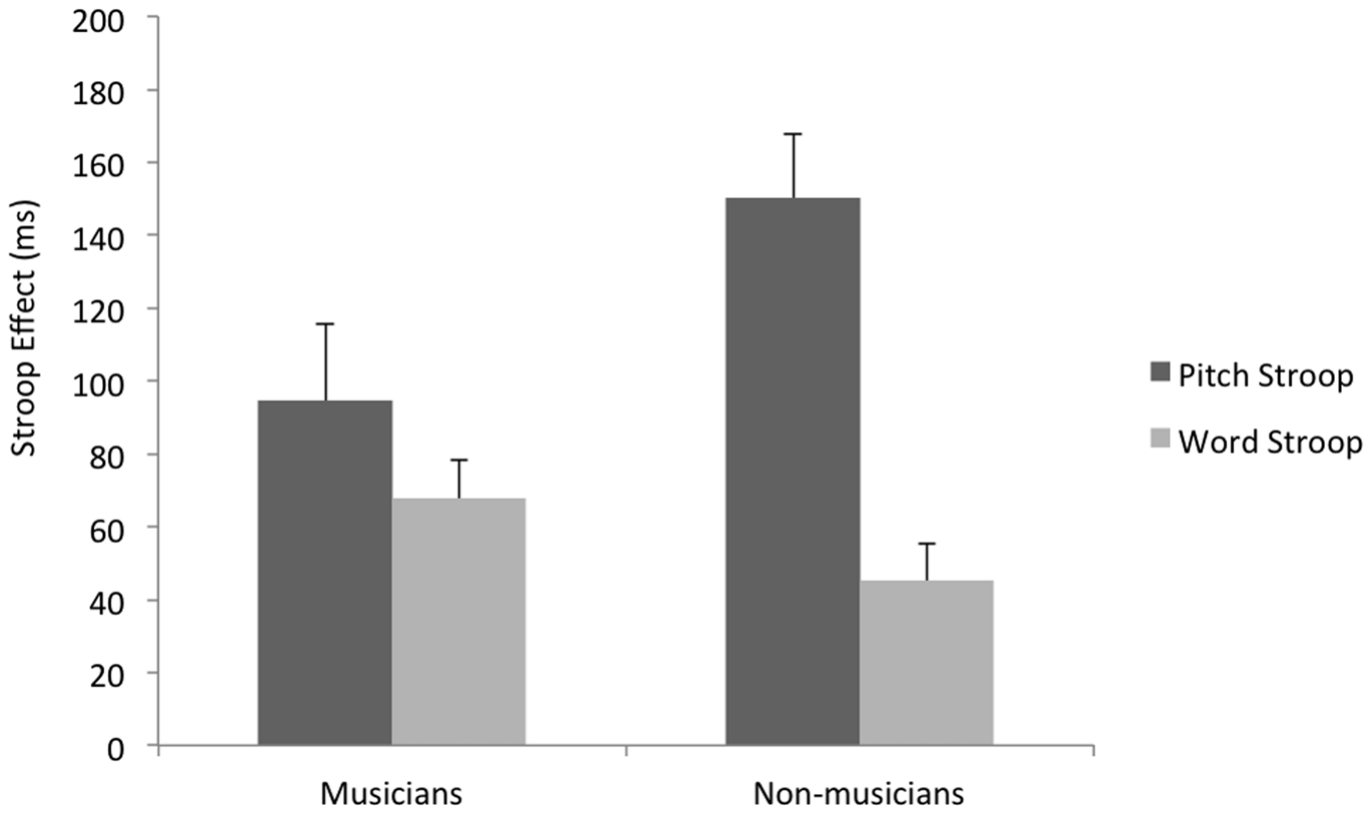
Mean Stroop effects for musicians and non-musicians on the pitch-conflict and word-conflict conditions. Musicians showed similar Stroop effects for the pitch and word conflict conditions, but non-musicians showed a significantly larger Stroop effect for the pitch condition compared to the word condition. Error bars are standard errors.

A final two-way ANOVA compared Stroop effects for musicians and non-musicians on pitch- and word-conflict conditions. There was a main effect of Condition, *F* (1, 39) = 19.71, *p*<.0001, η*_p_* ^2^ = .34, with word-conflict Stroop effects smaller than pitch conflict, and no effect of Group, *F* (1, 39) = 1.06, *p*>.3, η*_p_* ^2^ = .03, but there was a significant interaction between Condition and Group, *F* (1, 39) = 6.98, *p*<.05, η*_p_* ^2^ = .15. As illustrated in Figure 1, musicians’ Stroop effects for word and pitch identification did not differ, *p*>.2. Non-musicians, however, showed substantially larger Stroop effects for pitch than for words, *t*(22) = 5.35, *p*<.0001, *d* = 1.53. For these participants, conflicting words disrupted pitch identification, but conflicting pitch had little effect on word identification. Whereas non-musicians were perceptually biased toward words, musicians were perceptually flexible, ignoring words or pitch in accordance with task demands.

### Simon Task

RT comparisons for congruent and incongruent trials of the Simon task showed a main effect of Congruency, *F* (1, 40) = 48.70, *p*<.0001, η*_p_* ^2^ = .55, no effect of Group, *F* (1, 40) = 2.55, *p*>.1, η*_p_* ^2^ = .06, and an interaction between Congruency and Group, *F* (1, 40) = 13.56, *p*<.001, η*_p_* ^2^ = .25. Follow-up *t*-tests indicated that musicians were significantly faster than non-musicians on incongruent trials, *t*(40) = 2.70, *p*<.05, *d* = .84. Musicians and non-musicians showed reliable Simon effects, *p*s <.05, but musicians showed a smaller Simon effect, *t*(40) = 3.68, *p*<.001, *d* = 1.15. There were no differences on congruent trials, *p*>.8. Accuracy for both groups (*Mdn* = 93%) did not differ, *p*>.7.

### Visuospatial Span Task

Musicians had a significantly higher span score than non-musicians, *t*(39) = 2.81, *p*<.01, *d* = .89, indicating an advantage for musicians in visual working memory.

### Go/No-Go

Despite apparent differences favoring musicians for RTs on Go trials and rate of false alarms on No-Go trials, those differences were not statistically significant, *p*s ≥.15.

### Reading with Distraction

Reading task comparisons showed a significant effect of Condition, *F* (1, 39) = 141.01, *p*<.0001, η*_p_* ^2^ = .78, with faster reading of low-interference than high-interference stories. However, musicians’ small advantage in overall reading speed (*M* = 79.35 sec, *SD* = 20.18) relative to non-musicians (*M = *90.64 sec, *SD* = 29.76) fell short of statistical significance, *F* (1, 39) = 3.78, *p* = .059, η*_p_* ^2^ = .09. The interaction between Condition and Group was marginally significant, *F* (1, 39) = 4.00, *p* = .052, η*_p_* ^2^ = .09. Additional liberal analyses revealed that the groups did not differ in the low-interference condition, *p*>.1, but musicians were faster at reading stories in the high-interference condition, *t*(39) = 2.06 *p*<.05, *d* = .65. Evidence that musicians were less affected than non-musicians by distracting information was apparent in their errors: Musicians had fewer intrusions of irrelevant words, *t*(39) = 2.02, *p*<.05, *d* = .64, and fewer omissions of target words, *t*(39) = 2.26, *p*<.05, *d* = .72.

### Cognitive Control

To obtain a composite measure of cognitive control, scores on tasks or conditions involving conflict or interference resolution were submitted to a principal component analysis with varimax rotation of the factor structure. More specifically, scores on the Simon task, Reading with Distraction task, conflict conditions from the Auditory Stroop task, and the visuospatial span task were submitted to the analysis. We included the visuospatial span task (Corsi block task – a typical visual working memory measure) in the analysis because it has been shown to involve resolution of interference from past irrelevant sequences when presented in ascending order. Older adults’ visuospatial span on this task improves when interference is reduced through the use of visually distinct trials or when trials are presented in descending order [46], [52]. Moreover, performance on high interference visuospatial working memory tasks has been shown to be significantly correlated with executive control and higher-level cognition [53]. This correlation is stronger than visuospatial span tasks with low interference. For ease of interpretation and consistency across all tasks submitted to the analysis, span scores were transformed by subtracting from 100 for better performance to be indicated by lower values. To create single measures for each of the tasks with RT scores, scores on trials with no conflict were regressed out of the critical trials. Specifically, congruent trials were regressed out of incongruent trials in the Simon task and conflict conditions in the Auditory Stroop task, and reading time in the low-interference condition was regressed out of the high-interference condition in the Reading with Distraction task. Standardized residuals from each measure were used in the subsequent analysis. The number of false alarms from the Go/No-Go task did not correlate significantly with any of the other control tasks included, *p*s >.3, perhaps due to the relatively few No-Go errors by each group in our sample. This task was thus excluded from the analysis.

Two factors with eigenvalues above 1.00 emerged from the principal component analysis. The factor loadings on the rotated matrix are displayed in Table 3. Scores from the Corsi block task, Simon task, Reading with Distraction task, and pitch conflict condition from the Auditory Stroop task loaded on the first factor, which accounted for 37% of the variance (eigenvalue = 1.89). The word conflict condition from the Auditory Stroop task was the only measure to show a high factor loading on the second factor, which accounted for 21% of the variance (eigenvalue = 1.04). The factor analysis confirmed that the ability to identify a word in the face of a conflicting pitch differed from the other cognitive control abilities, which were represented by the first factor. Scores on the first principal component served as a measure of cognitive control and indicated that musicians (*M* = -0.60, *SD* = 0.79) had better cognitive control abilities than non-musicians (*M = *0.46, *SD* = 0.91), *t*(37) = 3.82, *p*<.001, *d* = 1.23. As expected, scores on the second principal component showed a numerical advantage for non-musicians (*M* = −0.15, *SD* = 0.95) compared to musicians (*M = *0.20, *SD* = 1.06), but the difference was not significant, *p*>.2.

**Table 3:** Factor loadings from principal component analysis.

Measure	Factor 1	Factor 2
Corsi Block	**.78**	.01
Simon	**.74**	.32
Reading with Distraction	**.54**	.07
Pitch Conflict	**.63**	−.39
Word Conflict	.10	**.90**

Note: Factor loadings above.5 are marked in boldface.

## Discussion

We asked whether high levels of music training and experience were associated with cognitive processing advantages in late middle-aged to older adults. To this end, we compared professional musicians with non-musicians matched on age, education, vocabulary, and general health on a near-transfer task and on several far-transfer tasks. The near-transfer task assessed speed of auditory processing and auditory conflict resolution, and the far-transfer tasks assessed visuospatial span, control over competing responses (Simon task), response inhibition (Go/No-Go), and control over distraction (Reading with Distraction). In general, musicians outperformed controls on the near-transfer task, consistent with various auditory processing advantages that have been behaviourally documented in young and older adults with high levels of music training [12], [33]–[34], [54]–[55]. This auditory advantage is also consistent with a recent neurophysiological study demonstrating that relative to non-musicians, older musicians show enhanced attention-dependent neural activity associated with isolating simultaneously occurring sounds [56]. However, musicians were not uniformly better than non-musicians on all aspects of auditory processing. Although musicians responded faster than non-musicians on pitch identification, as expected, they did not respond faster on word identification. Musicians showed similar control abilities whether they were ignoring pitch or ignoring words. Non-musicians, however, showed a non-significant trend towards a better ability to ignore pitch when identifying conflicting words (e.g., the word “high” presented at low pitch) relative to musicians, but they identified the pitch level more poorly in the face of conflicting words. The net result was that musicians outperformed non-musicians on pitch identification in the context of conflicting word cues, and non-musicians showed a non-significant advantage on word identification in the context of conflicting pitch cues. This performance pattern is consistent with the results found in a previous study on younger adults [12]. Thus, increased salience of pitch was advantageous for musicians when pitch was the target feature but disadvantageous when it served as distractor. It is important to note, however, that these results were based on self-reports of hearing function and should be interpreted with caution. Nevertheless, it seems reasonable to suggest that musicians’ advantage in auditory control is specific to pitch identification, given that group differences varied as a function of task condition, consistent with previous findings [12]. Moreover, if hearing function did differ between the groups, it would likely be in favour of the non-musicians, as musicians are at a greater risk of hearing loss due to cumulative noise exposure and fewer protective measures during the early years of training and practice [57].

On the far-transfer tasks, musicians showed advantages on visuospatial span and on multiple aspects of cognitive control, including conflict resolution in the spatial domain (Simon task) and control over irrelevant information while reading aloud. The advantage on cognitive control was further supported by musicians’ enhanced performance on a composite measure of cognitive control. The finding of enhanced spatial span in older musicians adds clarity to the inconsistent findings in the literature, which include a marginally significant span effect in one study [35] and no span effect in other studies involving the same task [36] or a different task [33]. The robust span effect in the present study, with its modest-sized sample of musicians, may be attributable to the use of professional musicians who had greater training and expertise than the amateur musicians in previous studies of visuospatial span in older adults [33], [35]–[36]. The visuospatial advantage may stem, in part, from professional musicians’ greater experience and proficiency with sight-reading and consequent memory enhancement for complex patterns. Goolsby [58] found, for example, that skilled sight-readers look farther ahead in a musical score than less skilled sight-readers, which implies that the former maintain longer visual sequences in working memory while performing. Undoubtedly, they also represent the sensorimotor sequences associated with the notation. It is not surprising, then, that professional musicians show structural and functional enhancement of brain regions involved in sight-reading and visuospatial processing [18]–[19], [41]. As noted, the spatial span measure in the present study, the Corsi block task, is vulnerable to interference from the recent past [46], [52]. The high skill level of the present sample of musicians may have reduced their vulnerability to interference from prior items, raising the possibility that older professional musicians have greater control over interfering memories than do their non-musician peers.

The remaining tasks were concerned with cognitive control or conflict resolution in non-auditory contexts. Musicians showed a smaller Simon effect than non-musicians, indicating a heightened ability to resolve spatial conflict. This finding, which may arise from musicians’ preserved visuospatial memory, is consistent with the enhanced visuospatial skills of orchestral musicians [19], [41]. Reading with distraction assessed older adults’ ability to control irrelevant visual and textual cues in the course of reading. Musicians were faster than non-musicians in the high-interference condition involving irrelevant words and made significantly fewer errors of omission (i.e., omitting relevant words) and intrusion (i.e., reading irrelevant words). On the Simon and reading tasks, musicians exercised better control than non-musicians over visually or spatially distracting events, suggesting preserved inhibitory regulation [49].

The single cognitive control task that failed to show reliable differences was the Go/No-Go task. This outcome, which is surprising in view of musicians’ greater ability to control conflict and resolve response competition in the Simon and Reading with Distraction tasks, may be attributable to the few false alarms by both groups. Alternatively, musicians’ ability to inhibit distracting information or prepotent responses may not be uniformly advantageous across contexts.

Taken together, the findings suggest that high levels of musical expertise and sustained engagement in music-making are associated with the enhancement or preservation of domain-general, cognitive control abilities in older adults. These findings are consistent with negative correlations of age with gray matter density in the left inferior frontal gyrus and with dorsolateral prefrontal cortex volume in non-musicians but not in orchestral musicians [19]. The findings also suggest that, similar to the effects of bilingualism on older adults’ general cognitive function [1]–[3], long-term music practice is one of the cognitively demanding activities that might moderate age-related cognitive decline. These shared effects may possibly be explained by improved neural efficiency of general control networks, allowing cognitively active individuals to better cope with age-related neural changes.

Our work adds to the growing evidence of enhanced cognitive control in musically trained young adults [12] and children [59]. On the one hand, our modest sample size can be construed as a limitation, especially in view of the conflicting findings across studies. On the other hand, the presence of differences despite the small sample is impressive. As noted, the professional status of musicians in the present study, in contrast to the amateur status of musicians in other studies of older adults [33], [35]–[36], may be of particular importance. However, it is also important to note that attaining professionalism, in general, may contribute to cognitive differences between the two groups.

The correlational design of the present study precludes claims of a causal role for intensive music training, but the cognitive impact of short-term musical interventions with older adults [37] lends credence to the possibility. Other factors such as social and economic advantages in early life may mediate some of the effects of long-term music training [28], [60]. The physical effort associated with regular practice and performance may also be relevant. For example, physical fitness interventions have demonstrable cognitive consequences in older adults, especially for tasks involving cognitive control [61]. Possible pre-existing personality differences, which may be correlated with cognitive ability, should also be taken into account [62]–[63]. However, findings demonstrating that personality does not completely explain the association between cognitive function and music experience in younger adults [62], and that bilinguals who typically do not differ from their monolingual counterparts in personality (or other pre-existing traits) also show cognitive advantages [1]–[3], [7], [64]–[67], suggest that music experience may enhance cognitive ability. Finally, the pleasure of performing in temporal synchrony with others is likely to have wide-ranging consequences for well-being and overall function [68].
